# The study of the role of insulin resistance as etiological factor in polycystic ovarian syndrome: a case control study

**DOI:** 10.1515/almed-2021-0098

**Published:** 2022-06-10

**Authors:** Jyoti R. Tilak, Anju Jain, Nishtha Wadhwa, H.R. Tilak, Ashok Kumar Ahirwar

**Affiliations:** Indian Council of Medical Research (ICMR), Delhi, India; Department of Biochemistry, Lady Hardinge Medical College, New Delhi, India; SRL Diagnostics, Gurugram, India; Bhandari Hospital and Research Center, Indore, MP, India; Department of Biochemistry, University College of Medical Sciences, New Delhi, 110095, India

**Keywords:** insulin resistance, normoglycemia, polycystic ovary syndrome (PCOS)

## Abstract

**Objectives:**

The relationship between insulin resistance (IR) and polycystic ovary syndrome (PCOS) has been consistently shown by several studies but what is the cause and what is the effect remained an unsolved issue. In recent years, IR has been suggested to be a key etiological factor which contributes to the severity of metabolic and reproductive features in PCOS. The aim of the present study is to determine the etiological role of IR in PCOS.

**Methods:**

This is an analytical case control study where 30 newly diagnosed normoglycemic cases of PCOS (according to Rotterdam revised criteria 2003) between the age group of 15 and 35 years were enrolled. A total of 30 age matched, apparently healthy women were selected from volunteers as controls. Fasting glucose was analysed by spectrophotometry and fasting insulin by chemiluminescence immunoassay. HOMA-IR, Log HOMA-IR, QUICKI, G/I ratio and FIRI were calculated using standard formulae.

**Results:**

The anthropometric parameters and markers of IR were high and QUICKI & G/I ratio were low in cases as compared to controls (p<0.05). Cases with BMI≥25 showed significantly higher IR markers and lower QUICKI & G/I ratio than BMI<25 cases and BMI matched controls. No significant difference was present in IR markers between high and low central obesity cases.

**Conclusions:**

The findings of our study suggest that in normoglycemic PCOS women, raised IR markers in obese patients cannot be attributed to obesity or central obesity alone. Presence of IR in newly diagnosed cases at such an early stage i.e., even before development of hyperglycemia and hyperinsulinemia suggest IR to be a causative factor in development of PCOS.

## Introduction

Polycystic ovary syndrome (PCOS) is the most common endocrine disorder in women of reproductive age group and has several clinical implications, presenting in up to 18% of this population [1, 2]. PCOS is characterized by hyperandrogenism, and evidence of ovarian dysfunction and/or micro-polycystic morphology of the ovary. However, there are other abnormalities seen, which are not included among the diagnostic features. In particular, a higher degree of insulin resistance (IR) is found in women with PCOS [1, 3]. Insulin resistance is defined as a state in which more than normal amount of insulin is required to elicit a response [4].

Several studies have indicated the presence of insulin resistance and compensatory hyperinsulinemia in approximately 80% of obese women with PCOS, and in 30–40% of lean women [5]. The mechanism of IR appears to be a post binding abnormality in insulin receptor-mediated signal transduction [6].

The relationship between insulin resistance and PCOS has been consistently shown by several studies but what is the cause and what is the effect still remains an unsolved issue [1]. The understanding of the origin and mechanisms of insulin resistance in PCOS is poor [7].

In recent years, IR has been suggested to be a key etiological factor which contributes to the severity of metabolic and reproductive features, with obesity known to exacerbate the clinical symptoms, in part by stimulating steroidogenesis and androgen production [8, 9].

PCOS is a challenging endocrine disorder to treat [9]. Lifestyle modifications are the corner stone for optimal treatment in women with PCOS. Weight reduction and exercise have been shown to improve menstrual disturbance and infertility in obese PCOS women. A reduction in central fat and improved IR has also shown improvement [10]. In overweight and obese patients, weight loss decreases serum insulin and androgen levels and reduces the risk of developing type 2 diabetes [11]. Hence, assessment of insulin sensitivity quantitatively may help in identification of patients at higher risk for metabolic sequelae like the development of diabetes and may also allow the selection of patients who are most likely to respond to treatment with insulin-sensitizing drugs [4]. Therefore, the aim of the present study was to find the etiological role of IR in PCOS.

## Materials and methods

This is an analytical case control study where 30 newly diagnosed normoglycemic (blood glucose <100 mg/dL) cases of PCOS (according to Rotterdam revised criteria 2003 [12] between the age group of 15 to 35 years were enrolled. Thirty age matched, apparently healthy women were selected from volunteers as controls. The study was conducted at gynaecology and adolescent clinic at Lady Hardinge Medical College and associated Hospitals between Nov 2012 and March 2014 after taking bilingual written informed consent from study subjects and ethical clearance from Institutional Ethical Committee. The present study was conducted according to Declaration of Helsinki. Women with related disorders like diabetes mellitus, hyperprolactinemia, androgen secreting tumours, adrenal hyperplasia and osteomalacia were excluded from the study.

Physical examination was performed and following anthropometric measurements were taken: height in cm, weight in kg, waist circumference in cm, hip circumference in cm, waist to hip ratio (WHR) calculated using formula: waist circumference (cm)/hip circumference (cm), body mass index (BMI) calculated using formula: weight (kg)/(height in m^2^)

Early morning fasting venous blood sample was collected on 2nd to 5th day of menstrual cycle and following parameters were analysed: Fasting glucose was analysed by spectrophotometry, fasting insulin (F. insulin) was analysed by chemiluminescence immunoassay.

– HOMA-IR (homoeostasis model assessment-IR) was calculated using formula: HOMA-IR = glucose (mg/dL) × insulin (µIU/mL)/405 [13].
– Log HOMA-IR was calculated by taking logarithm of HOMA-IR [13].
– QUICKI (quantitative insulin sensitivity check Index) was calculated using formula: 1/log Insulin (µIU/mL) + log glucose (mg/dL) [13].
– Glucose/insulin ratio (G/I ratio) [13].
– FIRI (fasting insulin resistance index) was calculated using formula: (fasting glucose × fasting insulin)/25 [13].

### Statistical analysis

The data generated from this study was analysed by using Microsoft excel 365 and SPSS software version 20. The results were expressed as mean ± SD (standard deviation). The data was checked for normality by applying Shapiro-Wilk test. The difference between the groups was evaluated by Student’s t-test and Mann-Whitney U test depending upon the normality. A two-tailed p-value less than 0.05 was considered statistically significant and less than 0.001 was considered highly significant.

## Results

The comparison of anthropometric parameters and IR markers between cases and controls are presented in Table 1. The anthropometric parameters and markers of IR were significantly higher and QUICKI & G/I ratio were significantly lower in cases than control group.

**Table 1:** Comparison of anthropometric parameters and IR markers between normoglycemic cases and controls.

Anthropometric and IR markers	Cases (n=30)	Controls (n=30)	p-Value
BMI	26.25 ± 5.32	23.36 ± 3.29	0.015^a^
WHR	0.861 ± 0.061	0.798 ± 0.060	<0.001^b^
Fasting Insulin	11.55 ± 10.80	5.63 ± 2.55	0.006^a^
HOMA-IR	2.47 ± 2.40	1.17 ± 0.53	0.007^a^
Log HOMA-IR	0.26 ± 0.33	0.03 ± 0.19	0.002^a^
QUICKI	3.12 ± 0.42	3.46 ± 0.50	0.006^a^
Glucose/insulin ratio	11.94 ± 7.19	18.31 ± 8.73	0.003^a^
FIRI (fasting insulin resistance index)	39.94 ± 38.89	18.97 ± 8.72	0.007^a^

^a^p-Value<0.05 was considered significant. ^b^p-Value<0.001 was considered highly significant.

Insulin resistance is significantly high in cases as compared to controls despite all of them being normoglycemic. It is not clear whether IR is because of high BMI or high central obesity (WHR) in cases or because of PCOS per se.

To assess this, 30 cases were divided into two groups based on BMI i.e., obese (BMI≥25) & lean (BMI<25) cases [14] and insulin resistance markers were compared between the two groups. As shown in Table 2, IR markers were higher and QUICKI & G/I ratio were lower in overweight and obese women than in lean women and the difference was statistically significant. This suggests IR strongly correlates with high BMI in PCOS cases. But to find out whether obesity alone is associated with IR or not, normoglycemic obese cases were compared with BMI matched controls. As seen in Table 3, cases with BMI≥25 showed significantly higher IR markers and lower QUICKI & G/I ratio than BMI matched controls which suggests that high IR in obese patients cannot be associated or attributed to obesity alone. Further to assess central obesity, the cases were divided into high WHR (**≥**0.85) and low WHR (<0.85) groups and IR markers were studied in them. Table 4 shows that no significant difference was present in IR markers between high and low central obesity cases. This suggests that central obesity might not be associated with the development of IR in PCOS.

**Table 2:** Comparison of IR markers in obese and lean normoglycemic cases.

IR markers	BMI≥25 (n=16)	BMI<25 (n=14)	p-Value
Fasting Insulin	15.23 ± 13.25	7.35 ± 4.73	0.039^a^
HOMA-IR	3.27 ± 0.2.93	1.54 ± 1.10	0.041^a^
Log HOMA-IR	0.385 ± 0.33	0.108 ± 0.26	0.018^a^
QUICKI	2.96 ± 0.31	3.29 ± 0.47	0.038^a^
Glucose/insulin ratio	9.33 ± 6.07	14.92 ± 7.41	0.034^a^
FIRI (fasting insulin resistance index)	53.01 ± 47.49	24.97 ± 17.86	0.041^a^

^a^p-Value<0.05 was considered significant.

**Table 3:** Comparison of IR markers between obese normoglycemic cases and BMI matched controls.

IR markers	CASES (n=16)	Controls (n=10)	p-Value
Fasting Insulin	15.23 ± 13.25	5.67 ± 3.56	0.014^a^
HOMA-IR	3.27 ± 2.93	1.21 ± 0.78	0.016^a^
Log HOMA-IR	0.39 ± 0.336	0.01 ± 0.24	0.004^a^
QUICKI	2.96 ± 0.31	3.51 ± 0.45	0.005^a^
Glucose/insulin ratio	9.33 ± 6.07	19.16 ± 7.92	0.004^a^
FIRI (fasting insulin resistance index)	53.03 ± 47.49	19.64 ± 12.74	0.016^a^

^a^p-Value<0.05 was considered significant.

**Table 4:** Comparison of IR markers between high and low central obesity cases.

IR markers	WHR≥0.85 (n=18)	WHR<0.85 (n=12)	p-Value
Fasting Insulin	11.85 ± 7.60	11.11 ± 14.77	0.874
HOMA-IR	2.52 ± 1.77	2.39 ± 3.21	0.904
Log HOMA-IR	0.303 ± 0.30	0.19 ± 0.37	0.375
QUICKI	3.02 ± 0.32	3.26 ± 0.53	0.187
Glucose/insulin ratio	10.28 ± 6.08	14.43 ± 8.24	0.151
FIRI (fasting insulin resistance index)	40.75 ± 28.76	38.72 ± 52.02	0.903

## Discussion

In our study we found that IR was significantly higher in PCOS cases than controls despite being normoglycemic, suggesting a major role of IR in pathogenesis of PCOS. Similar study done by Gao et al. in Chinese population concluded that PCOS patients with even normal glucose tolerance are more insulin resistant than controls [15].

In PCOS, the “central paradox” is that despite systemic insulin resistant state, ovary remains sensitive to insulin action to produce androgens.The number and affinity of insulin receptors have been found to be optimal in different insulin target tissues and ovary, and no structural and mutational abnormalities of insulin receptors could be detected in the PCOS women [16]. Thus, a post-receptor binding defect in the insulin signalling pathway appears to play an important role in the aetiology of selective insulin resistance [6, 17].

Various studies have shown hyperinsulinemia and IR as core mechanism in pathogenesis of PCOS [4, 10]. but only a few studies have been done which point towards aetiology of PCOS [7, 8].

On comparing obese with lean cases IR was found to be significantly higher in obese individuals which suggested strong correlation of obesity with the degree of IR. Hence, we further compared obese cases with BMI matched controls and IR was significantly increased in cases. Absence of IR in BMI matched obese controls suggests that obesity might not be solely responsible for IR. Studies done by K.H. Park et al. and D.J. Evans et al. also demonstrated similar results [14, 18, 19].

On assessing central obesity no relationship was found between high central obesity cases and IR. This suggests central obesity has no possible role in development of IR in newly diagnosed PCOS cases. Studies done by Park et al. and Evans et al. also stated that Insulin sensitivity is only partly related to differences in WHR i.e., central obesity rather than in overall adiposity [18, 19].

The presence of IR in newly diagnosed PCOS cases suggest that IR plays an important role in aetiology of PCOS. Hence, early assessment of IR should be done even in normoglycemic PCOS women so that measures of improving glucose metabolism like weight reduction, regular exercise, diet, and other lifestyle modifications can be taken at an early stage [15, 20].

### Limitations

One of the major drawbacks of our study is small sample size. A convenient sample size was taken due to the constraint of short time period of the study. Only surrogate markers of IR were used as opposed to the gold standard method for quantifying insulin sensitivity which is hyperinsulinemia euglycemic glucose clamp method. We did not determine the HbA_1c_ levels in the present study. Follow up study could be done to assess cause, mechanism, and molecular basis of IR in these patients.

## Conclusions

The findings of our study suggest that in normoglycemic PCOS women, IR markers are significantly increased in cases as compared to their age and BMI matched controls. The IR in these patients cannot be attributed to obesity or central obesity alone. These are newly diagnosed and normoglycemic cases, IR is present in them at such an early stage of the disease even before derangement of blood glucose and insulin. These findings suggest that IR must be present in them much before the development of PCOS. This implies IR to be a causative factor in development of PCOS.
